# 4,7,13,18-Tetra­oxa-1,10-diazo­nia­bicyclo­[8.5.5]icosane bis­(hexa­fluorido­phosphate)

**DOI:** 10.1107/S1600536811025992

**Published:** 2011-07-06

**Authors:** Nalinava Sen Gupta, David S. Wragg, Mats Tilset, Jon Petter Omtvedt

**Affiliations:** a Centre for Accelerator Based Research and Energy Physics (SAFE), Department of Chemistry, University of Oslo, PO Box 1038 Blindern, Oslo 0318, Norway; b inGAP Centre for Research Based Innovation, Center for Materials Science and Nanotechnology, Department of Chemistry, University of Oslo, PO Box 1033 Blindern, Oslo 0315, Norway; c Department of Chemistry, University of Oslo, PO Box 1033 Blindern, Oslo 0315, Norway

## Abstract

The asymmetric unit of the title structure, C_14_H_30_N_2_O_4_^2+^·2PF_6_^−^, contains the anion and half of the cation, the latter being completed by a crystallographic twofold axis. The cation has a cage structure with the ammonium H atoms pointing into the cage. These H atoms are shielded from inter­molecular inter­actions and form only intra­molecular contacts. There are short inter­molecular C—H⋯F inter­actions in the structure, but no conventional inter­molecular hydrogen bonds.

## Related literature

For related structures, see: Cos *et al.* (1982 ▶); Rehder & Wang (2003 ▶); Luger *et al.* (1991 ▶); Sen Gupta *et al.* (2011 ▶). For discussion of a cryptand as a mol­ecular automatic titrator, see: Alibrandi *et al.* (2009 ▶). For NMR data, see: Macchioni *et al.* (2001 ▶); Christe & Wilson (1990 ▶).
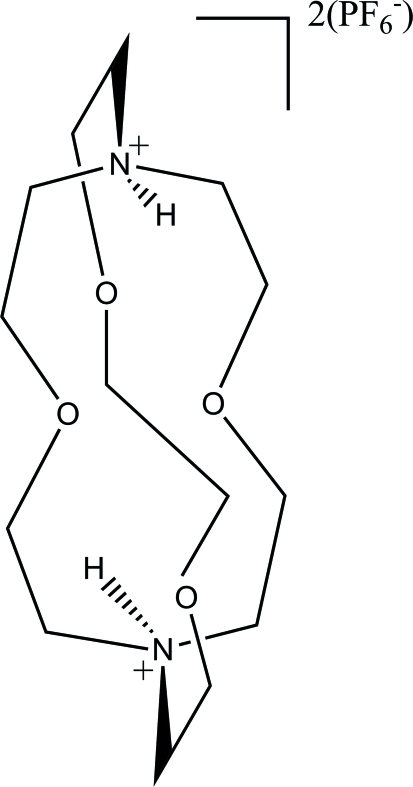


## Experimental

### 

#### Crystal data

C_14_H_30_N_2_O_4_^2+^·2PF_6_^−^
*M* *_r_* = 580.34
Monoclinic, 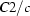

*a* = 10.8297 (16) Å
*b* = 16.485 (2) Å
*c* = 12.6846 (19) Å
β = 95.538 (2)°
*V* = 2254.0 (6) Å^3^
*Z* = 4
Mo *K*α radiation
μ = 0.32 mm^−1^
*T* = 296 K
0.24 × 0.06 × 0.02 mm

#### Data collection

Bruker SMART CCD area-detector diffractometer
Absorption correction: multi-scan (*SADABS*; Sheldrick, 2004 ▶) *T*_min_ = 0.977, *T*_max_ = 0.994
9756 measured reflections
2785 independent reflections
2002 reflections with *I* > 2σ(*I*)
*R*_int_ = 0.039

#### Refinement

*R*[*F* ^2^ > 2σ(*F* ^2^)] = 0.040
*wR*(*F* ^2^) = 0.095
*S* = 1.03
2785 reflections
154 parameters
H-atom parameters constrained
Δρ_max_ = 0.40 e Å^−3^
Δρ_min_ = −0.34 e Å^−3^

### 

Data collection: *SMART* (Bruker, 2001 ▶); cell refinement: *SAINT* (Bruker, 2001 ▶); data reduction: *SAINT*; program(s) used to solve structure: *SHELXS97* (Sheldrick, 2008 ▶); program(s) used to refine structure: *SHELXL97* (Sheldrick, 2008 ▶) and *WinGX* (Farrugia, 1999 ▶); molecular graphics: *DIAMOND* (Brandenburg & Berndt, 1999 ▶); software used to prepare material for publication: *publCIF* (Westrip, 2010 ▶).

## Supplementary Material

Crystal structure: contains datablock(s) I, global. DOI: 10.1107/S1600536811025992/fy2005sup1.cif

Structure factors: contains datablock(s) I. DOI: 10.1107/S1600536811025992/fy2005Isup2.hkl

Additional supplementary materials:  crystallographic information; 3D view; checkCIF report

## Figures and Tables

**Table 1:** Hydrogen-bond geometry (Å, °)

*D*—H⋯*A*	*D*—H	H⋯*A*	*D*⋯*A*	*D*—H⋯*A*
N1—H0⋯O1	0.91	2.34	2.826 (2)	113
N1—H0⋯O2	0.91	2.18	2.699 (2)	115
N1—H0⋯O1^i^	0.91	2.33	2.790 (2)	111
C2—H2*A*⋯F2^ii^	0.97	2.43	3.405 (2)	178
C6—H6*B*⋯F5	0.97	2.48	3.157 (2)	126

Symmetry codes: (i) 
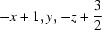
; (ii) 
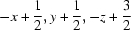
.

**Fig. 1.**
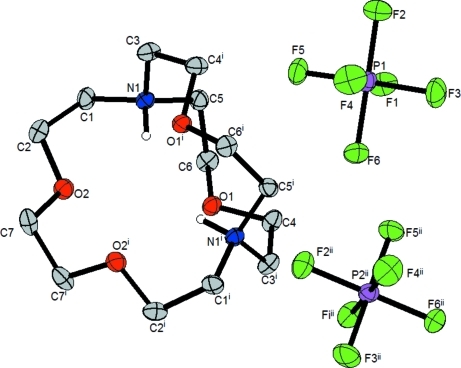
The molecular structure of (I), with atom labels and 50% probability displacement ellipsoids for non-H atoms. The hydrogen atoms attached to the carbons are omitted for clarity. Symmetry codes: (i) –x + 1, y, –z + 3/2.

**Fig. 2.**
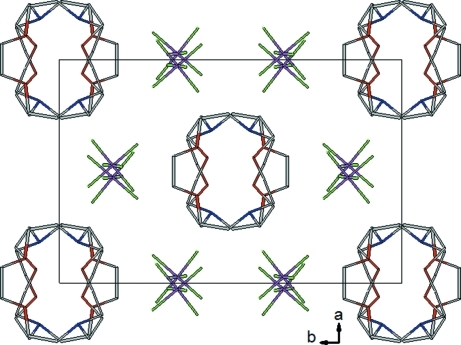
Packing diagram for (I), viewed along the c axis. H atoms are omitted for clarity.

## supplementary crystallographic information

### Comment

Compound (I) was obtained unintentionally as the product of the attempted synthesis of a metal-encrypted tungsten(VI) complex with the [2.1.1]cryptand, 4,7,13,18-tetraoxa-1,10-diazabicyclo[8.5.5]icosane. We suspect that WCl_6_, being susceptable to hydrolysis, reacted with water that was present as a contaminant. Compound (I) was obtained by recrystallisation of the crude reaction product from methanol. When the same product was recrystalised from acetone, a similar diprotonated cryptand salt with SiF_6_^2-^ as the anion formed (Sen Gupta *et al.*, 2011). The solvent used for recystallization was the only difference between the methods to obtain the two different crystals. The presence of both anions in the reaction product was confirmed by ^19^F NMR data (Macchioni *et al.*, 2001; Christe & Wilson, 1990).

In the crystal of compound (I), the two ammonium hydrogen atoms of the diprotonated cryptand cage are pointing inwards. Cryptands are known to form proton crypts, in which the protons are very efficiently concealed inside a tight molecular cavity. No exception is observed here: the ammonium hydrogen atoms are not involved in intermolecular hydrogen bonding. They only form intramolecular contacts with the oxygen atoms of the cryptand.

### Experimental

Reagents were purchased from Sigma-Aldrich and were used without further purification. Reactions were carried out under inert conditions by Schlenk-line techniques. The metal chloride (WCl_6_, 100 mg, 0.25 mmol) was allowed to stir for a minute in 10 ml toluene and then was reacted with a small excess of of AgPF_6_ (381 mg, 1.51 mmol) to give AgCl as a precipitate and W(PF_6_)_6_ dissolved in solution. After 30 minutes stirring, the precipitate was allowed to settle. The solution was transferred under inert conditions by cannula technique and treated with the solution of [2.1.1]cryptand (66 µl, 0.25 mmol) in 5 ml toluene for 30 minutes. The crude reaction product was obtained as dirty yellow mass after drying the solvent and it was found to be soluble in methanol. Portions of the product were recrystallized from methanol, which produced crystal (I).

### Refinement

Hydrogen *U*_iso_'s were set at 1.2 times the *U*_eq_ of the heavy atom to which the hydrogen was attached and refined in riding mode. C—H distances were fixed at 0.97 Å and the N—H distance at 0.91 Å.

### Crystal data

**Table:**

C_14_H_30_N_2_O_4_^2+^·2PF_6_^−^	*F*(000) = 1192
*M**_r_* = 580.34	*D*_x_ = 1.710 Mg m^−^^3^
Monoclinic, *C*2/*c*	Mo *K*α radiation, λ = 0.71073 Å
Hall symbol: -C 2yc	Cell parameters from 1782 reflections
*a* = 10.8297 (16) Å	θ = 2.3–28.9°
*b* = 16.485 (2) Å	µ = 0.32 mm^−^^1^
*c* = 12.6846 (19) Å	*T* = 296 K
β = 95.538 (2)°	Needle, colourless
*V* = 2254.0 (6) Å^3^	0.24 × 0.06 × 0.02 mm
*Z* = 4	

### Data collection

**Table:**

Bruker SMART CCD area-detector diffractometer	2785 independent reflections
Radiation source: sealed tube	2002 reflections with *I* > 2σ(*I*)
graphite	*R*_int_ = 0.039
φ and ω scans	θ_max_ = 28.9°, θ_min_ = 2.3°
Absorption correction: multi-scan (*SADABS*; Sheldrick, 2004)	*h* = −14→14
*T*_min_ = 0.977, *T*_max_ = 0.994	*k* = −22→21
9756 measured reflections	*l* = −17→16

### Refinement

**Table:**

Refinement on *F*^2^	Primary atom site location: structure-invariant direct methods
Least-squares matrix: full	Secondary atom site location: difference Fourier map
*R*[*F*^2^ > 2σ(*F*^2^)] = 0.040	Hydrogen site location: inferred from neighbouring sites
*wR*(*F*^2^) = 0.095	H-atom parameters constrained
*S* = 1.03	*w* = 1/[σ^2^(*F*_o_^2^) + (0.0378*P*)^2^ + 1.6619*P*] where *P* = (*F*_o_^2^ + 2*F*_c_^2^)/3
2785 reflections	(Δ/σ)_max_ = 0.001
154 parameters	Δρ_max_ = 0.40 e Å^−^^3^
0 restraints	Δρ_min_ = −0.34 e Å^−^^3^

### Special details

**Table:**

Geometry. All s.u.'s (except the s.u. in the dihedral angle between two l.s. planes) are estimated using the full covariance matrix. The cell s.u.'s are taken into account individually in the estimation of s.u.'s in distances, angles and torsion angles; correlations between s.u.'s in cell parameters are only used when they are defined by crystal symmetry. An approximate (isotropic) treatment of cell s.u.'s is used for estimating s.u.'s involving l.s. planes.
Refinement. Refinement of *F*^2^ against ALL reflections. The weighted *R*-factor *wR* and goodness of fit *S* are based on *F*^2^, conventional *R*-factors *R* are based on *F*, with *F* set to zero for negative *F*^2^. The threshold expression of *F*^2^ > 2σ(*F*^2^) is used only for calculating *R*-factors(gt) *etc*. and is not relevant to the choice of reflections for refinement. *R*-factors based on *F*^2^ are statistically about twice as large as those based on *F*, and *R*- factors based on ALL data will be even larger.

### Fractional atomic coordinates and isotropic or equivalent isotropic displacement parameters (Å^2^)

**Table:**

	*x*	*y*	*z*	*U*_iso_*/*U*_eq_	
P1	0.47273 (5)	0.15573 (3)	0.99505 (4)	0.02088 (14)	
O1	0.56230 (12)	0.43242 (8)	0.89074 (10)	0.0200 (3)	
F1	0.46836 (11)	0.12944 (8)	1.11636 (9)	0.0315 (3)	
F2	0.35902 (11)	0.09625 (8)	0.96176 (9)	0.0316 (3)	
O2	0.39311 (12)	0.59898 (8)	0.80510 (11)	0.0222 (3)	
F6	0.58658 (11)	0.21576 (7)	1.02955 (10)	0.0334 (3)	
F5	0.37765 (11)	0.22713 (7)	1.01299 (11)	0.0366 (3)	
F3	0.56899 (12)	0.08506 (8)	0.98002 (11)	0.0379 (3)	
F4	0.47690 (13)	0.18356 (9)	0.87520 (10)	0.0421 (4)	
N1	0.31687 (14)	0.44308 (9)	0.79332 (12)	0.0180 (3)	
H0	0.3874	0.4684	0.7775	0.022*	
C6	0.46254 (18)	0.40126 (12)	0.94552 (15)	0.0217 (4)	
H6A	0.4339	0.4429	0.9914	0.026*	
H6B	0.4917	0.3556	0.9893	0.026*	
C5	0.35700 (18)	0.37461 (12)	0.86707 (15)	0.0203 (4)	
H5A	0.3831	0.3289	0.8265	0.024*	
H5B	0.2877	0.3573	0.9046	0.024*	
C4	0.65712 (18)	0.37384 (12)	0.87734 (16)	0.0225 (4)	
H4A	0.6216	0.3256	0.8426	0.027*	
H4B	0.6988	0.3583	0.9455	0.027*	
C1	0.24085 (18)	0.50610 (12)	0.84409 (16)	0.0218 (4)	
H1A	0.1536	0.4921	0.8331	0.026*	
H1B	0.2648	0.5079	0.9197	0.026*	
C3	0.25267 (18)	0.41362 (13)	0.69006 (15)	0.0227 (4)	
H3A	0.2131	0.4591	0.6518	0.027*	
H3B	0.1887	0.3750	0.7040	0.027*	
C7	0.43045 (19)	0.66749 (12)	0.74776 (17)	0.0257 (5)	
H7A	0.4019	0.7170	0.7788	0.031*	
H7B	0.3947	0.6646	0.6747	0.031*	
C2	0.26190 (18)	0.58770 (12)	0.79590 (16)	0.0238 (4)	
H2A	0.2286	0.5888	0.7221	0.029*	
H2B	0.2222	0.6300	0.8336	0.029*	

### Atomic displacement parameters (Å^2^)

**Table:**

	*U*^11^	*U*^22^	*U*^33^	*U*^12^	*U*^13^	*U*^23^
P1	0.0180 (3)	0.0221 (3)	0.0230 (3)	0.0016 (2)	0.0041 (2)	0.0031 (2)
O1	0.0164 (7)	0.0203 (7)	0.0237 (7)	0.0008 (5)	0.0041 (5)	0.0034 (6)
F1	0.0293 (7)	0.0440 (8)	0.0210 (6)	−0.0057 (6)	0.0018 (5)	0.0048 (5)
F2	0.0312 (7)	0.0350 (7)	0.0281 (7)	−0.0101 (6)	−0.0007 (5)	−0.0018 (5)
O2	0.0215 (7)	0.0205 (7)	0.0249 (7)	0.0003 (6)	0.0034 (6)	0.0057 (6)
F6	0.0221 (6)	0.0305 (7)	0.0476 (8)	−0.0053 (5)	0.0031 (6)	0.0034 (6)
F5	0.0252 (7)	0.0270 (7)	0.0587 (9)	0.0080 (5)	0.0097 (6)	0.0028 (6)
F3	0.0338 (7)	0.0298 (7)	0.0523 (9)	0.0105 (6)	0.0158 (6)	−0.0002 (6)
F4	0.0452 (8)	0.0555 (9)	0.0260 (7)	−0.0063 (7)	0.0063 (6)	0.0133 (6)
N1	0.0143 (8)	0.0194 (8)	0.0208 (9)	−0.0003 (6)	0.0039 (6)	−0.0006 (7)
C6	0.0219 (10)	0.0252 (11)	0.0186 (10)	−0.0007 (8)	0.0047 (8)	0.0038 (8)
C5	0.0198 (10)	0.0182 (10)	0.0233 (10)	0.0004 (8)	0.0045 (8)	0.0029 (8)
C4	0.0197 (10)	0.0249 (11)	0.0228 (11)	0.0042 (8)	0.0012 (8)	0.0048 (8)
C1	0.0197 (10)	0.0212 (10)	0.0256 (11)	0.0047 (8)	0.0077 (8)	−0.0024 (8)
C3	0.0174 (10)	0.0281 (11)	0.0221 (10)	−0.0032 (8)	−0.0003 (8)	−0.0028 (8)
C7	0.0339 (12)	0.0160 (10)	0.0286 (11)	0.0049 (8)	0.0102 (9)	0.0021 (8)
C2	0.0205 (10)	0.0270 (11)	0.0240 (11)	0.0061 (8)	0.0030 (8)	0.0014 (8)

### Geometric parameters (Å, °)

**Table:**

P1—F3	1.5871 (13)	C5—H5A	0.9700
P1—F4	1.5927 (13)	C5—H5B	0.9700
P1—F5	1.5946 (13)	C4—C3^i^	1.509 (3)
P1—F2	1.5987 (13)	C4—H4A	0.9700
P1—F1	1.6037 (13)	C4—H4B	0.9700
P1—F6	1.6083 (13)	C1—C2	1.504 (3)
O1—C4	1.432 (2)	C1—H1A	0.9700
O1—C6	1.435 (2)	C1—H1B	0.9700
O2—C7	1.423 (2)	C3—C4^i^	1.509 (3)
O2—C2	1.427 (2)	C3—H3A	0.9700
N1—C3	1.503 (2)	C3—H3B	0.9700
N1—C5	1.503 (2)	C7—C7^i^	1.502 (4)
N1—C1	1.508 (2)	C7—H7A	0.9700
N1—H0	0.9100	C7—H7B	0.9700
C6—C5	1.507 (3)	C2—H2A	0.9700
C6—H6A	0.9700	C2—H2B	0.9700
C6—H6B	0.9700		
			
F3—P1—F4	91.00 (8)	C6—C5—H5B	109.6
F3—P1—F5	178.59 (8)	H5A—C5—H5B	108.1
F4—P1—F5	90.10 (8)	O1—C4—C3^i^	106.57 (15)
F3—P1—F2	90.93 (7)	O1—C4—H4A	110.4
F4—P1—F2	90.95 (7)	C3^i^—C4—H4A	110.4
F5—P1—F2	89.93 (7)	O1—C4—H4B	110.4
F3—P1—F1	89.85 (7)	C3^i^—C4—H4B	110.4
F4—P1—F1	178.93 (8)	H4A—C4—H4B	108.6
F5—P1—F1	89.04 (7)	C2—C1—N1	109.40 (15)
F2—P1—F1	89.68 (7)	C2—C1—H1A	109.8
F3—P1—F6	89.38 (7)	N1—C1—H1A	109.8
F4—P1—F6	89.48 (7)	C2—C1—H1B	109.8
F5—P1—F6	89.75 (7)	N1—C1—H1B	109.8
F2—P1—F6	179.46 (8)	H1A—C1—H1B	108.2
F1—P1—F6	89.88 (7)	N1—C3—C4^i^	111.34 (16)
C4—O1—C6	113.47 (14)	N1—C3—H3A	109.4
C7—O2—C2	113.05 (15)	C4^i^—C3—H3A	109.4
C3—N1—C5	112.42 (15)	N1—C3—H3B	109.4
C3—N1—C1	111.66 (15)	C4^i^—C3—H3B	109.4
C5—N1—C1	112.86 (15)	H3A—C3—H3B	108.0
C3—N1—H0	106.5	O2—C7—C7^i^	108.31 (15)
C5—N1—H0	106.5	O2—C7—H7A	110.0
C1—N1—H0	106.5	C7^i^—C7—H7A	110.0
O1—C6—C5	110.07 (15)	O2—C7—H7B	110.0
O1—C6—H6A	109.6	C7^i^—C7—H7B	110.0
C5—C6—H6A	109.6	H7A—C7—H7B	108.4
O1—C6—H6B	109.6	O2—C2—C1	105.79 (15)
C5—C6—H6B	109.6	O2—C2—H2A	110.6
H6A—C6—H6B	108.2	C1—C2—H2A	110.6
N1—C5—C6	110.33 (16)	O2—C2—H2B	110.6
N1—C5—H5A	109.6	C1—C2—H2B	110.6
C6—C5—H5A	109.6	H2A—C2—H2B	108.7
N1—C5—H5B	109.6		
			
C4—O1—C6—C5	−96.35 (18)	C5—N1—C1—C2	149.57 (16)
C3—N1—C5—C6	156.50 (15)	C5—N1—C3—C4^i^	−71.3 (2)
C1—N1—C5—C6	−76.12 (19)	C1—N1—C3—C4^i^	160.72 (16)
O1—C6—C5—N1	−55.1 (2)	C2—O2—C7—C7^i^	−173.23 (18)
C6—O1—C4—C3^i^	174.66 (15)	C7—O2—C2—C1	170.30 (16)
C3—N1—C1—C2	−82.64 (19)	N1—C1—C2—O2	−53.0 (2)

Symmetry codes: (i) −*x*+1, *y*, −*z*+3/2.

### Hydrogen-bond geometry (Å, °)

**Table:**

*D*—H···*A*	*D*—H	H···*A*	*D*···*A*	*D*—H···*A*
N1—H0···O1	0.91	2.34	2.826 (2)	113
N1—H0···O2	0.91	2.18	2.699 (2)	115
N1—H0···O1^i^	0.91	2.33	2.790 (2)	111
C2—H2A···F2^ii^	0.97	2.43	3.405 (2)	178
C6—H6B···F5	0.97	2.48	3.157 (2)	126

Symmetry codes:  (i) −*x*+1, *y*, −*z*+3/2; (ii) −*x*+1/2, *y*+1/2, −*z*+3/2.
